# Stereotypy with Parkinsonism as a Rare Sequelae of Dengue Encephalitis: A Case Report and Literature Review

**DOI:** 10.5334/tohm.630

**Published:** 2021-06-23

**Authors:** V. H. Ganaraja, Nitish Kamble, M. Netravathi, Vikram V. Holla, Neeraja Koti, Pramod Kumar Pal

**Affiliations:** 1 Department of Neurology, National Institute of Mental Health and Neuro Sciences (NIMHANS), Hosur Road, Bangalore-560029, Karnataka, India

**Keywords:** Dengue encephalitis, Encephalitic sequelae, Parkinsonism, Snapping movements, Stereotypy, Viral encephalitis

## Abstract

**Background:**

Parkinsonism following viral encephalitis is well reported. However, in addition, to parkinsonism other movement disorders such as dystonia, chorea, myoclonus may also be observed in these patients. Stereotypy is a very rare manifestation following viral encephalitis.

**Case report:**

Here we report a rare case of a 25-year-old young man who developed stereotypy and parkinsonism following dengue virus encephalitis. The stereotypy was in the form of snapping of fingers of left-hand which was repetitive, purposeless, non-goal directed, present for most of the day and partially suppressible.

**Discussion:**

This report expands the spectrum of movement disorders seen in dengue infection.

## Introduction

Post encephalitic parkinsonism was initially described following an influenza pandemic in 1917 which was later infrequently reported [1]. Stereotypy has been described before as a part of various neurodegenerative disorders such as Rett syndrome, neuroacanthocytosis and also in autoimmune disorders such as PANDAS. However, stereotypy following dengue virus encephalitis has not been reported till date. Here we describe a rare case of stereotypy with parkinsonism following dengue virus encephalitis. The patient and his guardians have provided a written informed consent for publishing the clinical details including the video.

## Case Report

About 4 months back, a 25-year-old young man had high-grade fever, headache and vomiting for 5 days and later developed altered sensorium. He was admitted in the intensive care unit of a nearby hospital for 10 days. Investigations revealed a positive dengue NS1 antigen test. He was treated symptomatically and over the next 15 days, the sensorium gradually improved. During the recovery phase, the patient was found to have dysarthria and reduced speech output. Two months following encephalitis, he developed slowness while walking and a feeling of stiffness in both lower limbs. He required one-person support to walk and had toe walking with bent knees. In addition, he developed snapping of fingers of left-hand which was repetitive, purposeless and non-goal directed. It was present for most of the day and was partially suppressible. There was no feeling of discomfort or urge to perform these movements on voluntary suppression. It was sometimes associated with tremulousness of left index finger. The patient was aware of the symptoms but could not control them completely. These movements would subside during sleep. There was no progression in the severity of these snapping movements till the time he presented to us.

He was born to a non-consanguineous parentage with normal birth and developmental history. There was no history of neurological illness, movement disorders (dystonia/parkinsonism) or psychiatric illness in the family. There was no history of psychiatric illness in the past and he was never treated with dopamine blockers or other medications. There was no history of alcohol or substance abuse. Our patient hails from north Karnataka state in the southern part of India which is endemic for dengue. He was working in a grocery shop and there was no history of exposure to alcohol or chemicals/solvents.

On examination, the patient was conscious, alert and responsive to commands. His vital parameters were within normal limits. On neurological examination, he had mild up-gaze restriction along with jerky pursuits and normal saccades. He also had reduced facial expression. His speech was severely hypophonic with palilalia. Examination of other cranial nerves was normal. Paratonia was observed in both the upper limbs and spasticity in lower limbs. There was a mild head flexion to left with dystonic posturing of right hand. Hand grip of both sides were normal. Lower limb movements were restricted due to spasticity; however, he was able to lift against gravity. All deep tendon reflexes were brisk with bilateral extensor plantar responses. Sensory examination was normal.

He had repetitive, coordinated and patterned snapping movements involving the left thumb and middle finger which were partially suppressible. In addition, there was slow and coarse tremor of the left index finger (***Video segment 1***). Generalized bradykinesia was present along with micrographia. He had a stooped posture with knees flexed, severe freezing of gait and needed one-person support to walk, (***Video segment 2***). Other systemic examinations were unremarkable.

**Video:** **Video segment 1 Stereotypy involving left thumb and middle finger. Video segment 2: Bradykinesia with gait freezing and stooped posture**. **Segment 1** Involuntary, patterned and coordinated snapping movements observed involving left thumb and middle finger which is partially suppressible. **Segment 2** Bradykinesia involving upper and lower limbs along with snapping movements in left hand as described and severe gait freezing.

**Figure 1:**
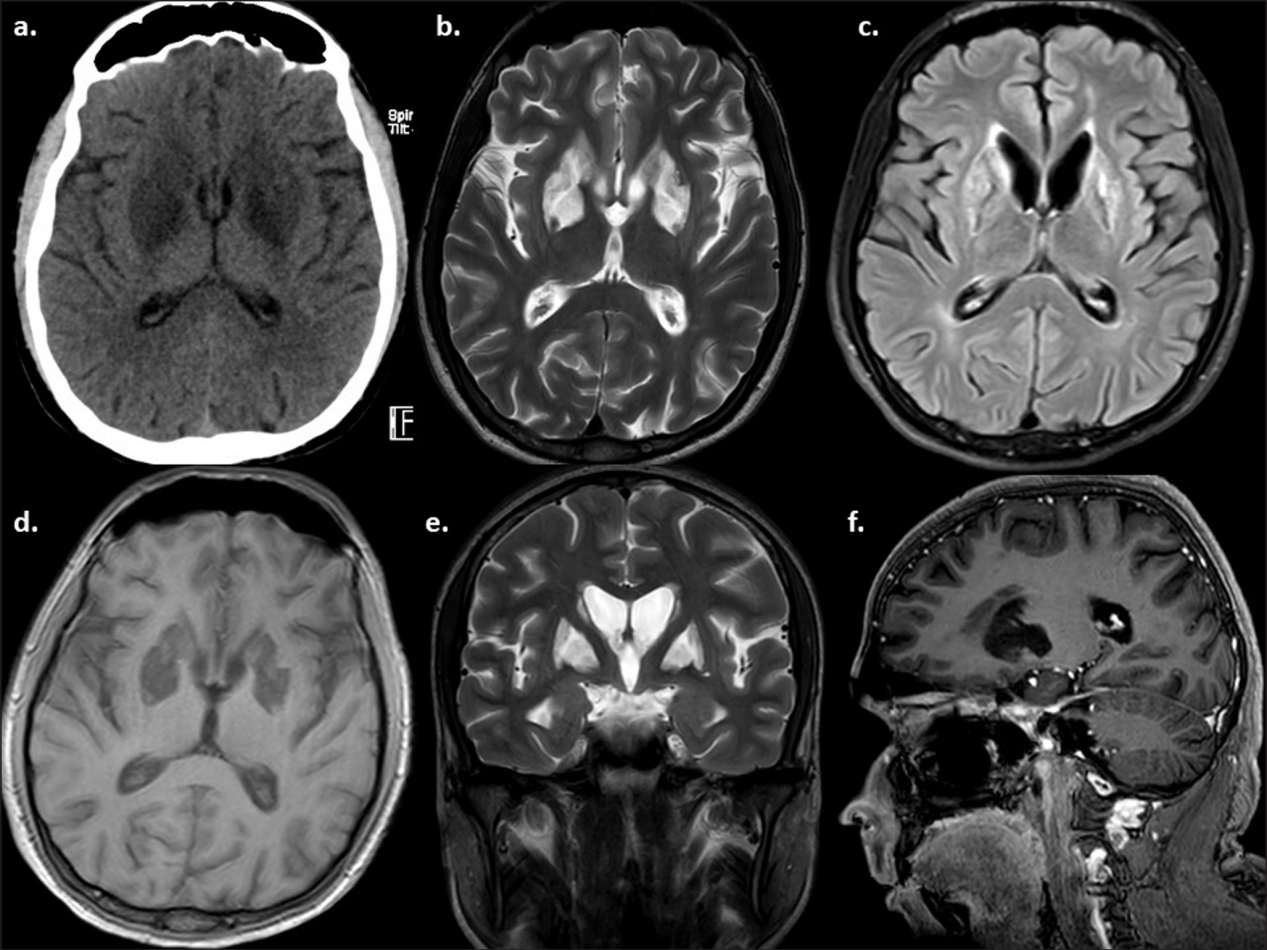
CT brain axial **(a)** showing hypo density in bilateral basal ganglia and MRI findings **(b)**-T2 Axial, **(c)**-FLAIR Axial, **(d)**-T1 Axial, **(e)**-T2 coronal sequences showing bilateral basal ganglia involvement. **(f)**-T1 coronal at the level of basal ganglia without any contrast enhancement.

His routine blood investigations- complete hemogram, liver and kidney function tests were normal. Serum IgM antibodies against dengue virus were detected. Antibodies against chickungunya and Japanese encephalitis infections were negative. Screening for HIV, Hepatitis B, hepatitis C and valuations for autoimmune encephalitis were negative. Serum copper/ceruloplasmin were within normal limits. CSF was acellular and normal protein and glucose. Ultrasound abdomen was normal. Brain MRI showed atrophy with bilateral basal ganglia T2/FLAIR hyperintensities without any contrast enhancement (***Figure 1***). He was treated symptomatically with combination of levodopa-carbidopa (400 mg/day), baclofen (30 mg/day), pramipexole (0.75 mg/day), amantadine (100 mg/day), tolperisone (50 mg/day) and diazepam (6 mg/day). In addition, the patient also underwent physiotherapy, neurorehabilitation and speech therapy. There was minimal improvement in parkinsonism symptoms with no improvement in stereotypy.

## Discussion

Here we report a combination of stereotypy and parkinsonism which is an unusual complication of dengue encephalitis. Stereotypy is an involuntary, coordinated, patterned repetitive, rhythmic and purposeless movement [2]. Commonly stereotypies are seen as arm flapping, finger wiggling, head nodding and body rocking, often associated with neurodegenerative conditions such as Rett syndrome, neuroacanthocytosis and sometimes even in normal individuals. Secondary stereotypies following encephalitis are very rare. Face scratching stereotypy has been described in herpetic encephalitis [3] and nose pinching in seronegative autoimmune encephalitis [4]. Rapid tremor like stereotyped movements involving right hand and foot is also reported in anti-basal ganglia antibody disease [5]. However stereotypic snapping movements of hand as a sequelae of dengue encephalitis have not been described till date. Our patient also had parkinsonism in addition to stereotypy. Though first description of delayed parkinsonism following encephalitis was in 1918 by Von Economo related to influenza pandemic [6], parkinsonism as a sequele to dengue encephalitis was described very recently [7].

There are only a handful of reports of various movement disorders following dengue encephalitis, common being parkinsonism, ataxia and opsoclonus-myoclonus syndrome (***Table 1***). The pathophysiology of these movement disorders following encephalitis is not well understood. Neurotropic mechanism, systemic complication and immune mediated damage have been hypothesized to cause neurological manifestations [8]. Extrapyramidal manifestations in viral encephalitis have been postulated to be due to involvement of basal ganglia, mainly substantia nigra by neurotropic viruses [9]. Cellular changes leading to basal ganglia destruction is due to formation of Lewy bodies and cell death in nigral region [10]. However, in encephalitis, inflammation effecting dopamine neurotransmission has been postulated to be one of the mechanisms for these movement disorders [11]. Inability to identify dengue virus in all the reported cases further strengthens the immune hypothesis as a mechanism for dengue encephalitis sequele in these patients. Post viral parkinsonism is usually refractory to anti-parkinsonian medications but may respond to immunosuppressants such as steroids, which again favor the role of immune mechanisms in this spectrum of disorders [12].

**Table 1:** Reports of various movement disorders following dengue encephalitis.


S.NO.	STUDY AND YEAR	COUNTRY	AGE	GENDER	MOVEMENT DISORDERS	BRAIN MRI	DENGUE IGM ANTIBODY(SERUM)	DENGUE IGG ANTIBODY(SERUM)	DENGUE NS1 ANTIGEN	DENGUE IGM ANTIBODY(CSF)	REFERENCE NO

1	Matta et al.2004	Brazil	10Yrs	Female	Diminished level of consciousness, spastic tetra paresis, cerebellar syndrome and frontal symptoms	Cerebral peduncle, lentiform nuclei and internal capsule signal changes on both sides	Diagnosis was made by ELISA	NA	NA	NA	[14]

2	Verma et al. 2011	India	34Yrs	Female	Opsoclonus Myoclonus Syndrome	Normal	Positive	NA	NA	NA	[15]

			19Yrs	Female	Opsoclonus Myoclonus Syndrome	Normal	positive	NA	NA	NA	

3	Karunarathne et al. 2012	Sri Lanka	43Yrs	Male	Ataxia	Hyperintesnity in bilateral cerebellar hemisphere spreading across vermis	Positive for Dengue and EBV	Positive	NA	NA	[16]

4	Azmin et al. 2013	Malaysia	18Yrs	Male	Parkinsonism with multiple cranial neuropathy with cerebellar ataxia and brachial plexopathy	Normal	Positive	Positive	Positive	NA	[7]

5	Withana et al. 2014	Sri Lanka	45Yrs	Female	Ataxia	Normal	Positive	Positive	Positive	NA	[17]

6	Fong et al. 2014	Malaysia	6Yrs	Female	Parkinsonism	Normal	Positive	NA	Positive	NA	[18]

7	Weeratunga et al. 2014	Sri Lanka	40Yrs	Female	Ataxia	Normal	Positive	NA	NA	Positive	[19]

			28Yrs	Male	Ataxia	Normal	Positive	NA	NA	Positive	

			25Yrs	Male	Ataxia	Bilateral and symmetrical T2 hyper intense lesions in the cerebellum	Positive	NA	NA	Positive	

8	Tan et al 2014	Malaysia	30Yrs	Male	Opsoclonus Myoclonus Syndrome	Pachy- and leptomeningeal enhancement.	Positive	Positive	NA	NA	[20]

			10Yrs	Male	Opsoclonus Myoclonus Syndrome	NA	Positive	NA	positive	NA	

9	Patel et al. 2017	India	16Yrs	Male	Ataxia	Signal intensity alteration in pons, medulla, superior, and middle cerebellar peduncles with patchy enhancement.	Positive	NA	NA	NA	[21]

10	Bopeththa et al. 2017	Sri Lanka	69Yrs	Male	Parkinsonism	Normal	Positive	Negative	Negative	Positive	[22]

11	Khoo et al. 2018	Malaysia	60Yrs	Male	Ataxia	Hyperintense signals at the right corona radiata and left frontal lobe (in keeping with old stroke).	Positive	NA	NA	NA	[23]

12	Desai et al. 2018	India	14Yrs	Male	Opsoclonus myoclonus	Normal	Positive	Positive	Positive	NA	[8]

13	Manapalli et al.2019	India	48Yrs	Male	Parkinsonism	Micro infarcts in the basal ganglia	Positive	NA	Positive	NA	[12]

14	Panda et al. 2020	India	13Yrs	Male	Parkinsonism	Normal	NA	NA	Positive	NA	[24]

15	Dudipala et al 2020	India	11Yrs	Female	Myoclonus	Normal	Positive	NA	NA	NA	[25]

16	Mishra et al. 2020	India	18Yrs	Female	Generalized dystonia/Parkinsonism	Double Doughnut sign	NA	NA	Positive	NA	[26]

17	Current study	India	25 Yrs	Male	Stereotypy with parkinsonism	Bilateral basal ganglia T2/FLAIR hyper intensities	Positive	NA	Positive	NA	


Abbreviations: NA-Not Available, Yrs-Years.

In our case, the presence of fever with acute signs of cerebral involvement and presence of IgM dengue antibodies in serum, which can persist up to 3 months after febrile phase, meets the criteria for dengue encephalitis [13]. We also ruled out other causes of encephalitis by appropriate investigations. This report expands the spectrum of movement disorders seen in dengue infection and is the first report of stereotypy-parkinsonism following dengue virus.
